# Decision Making in the Management of Extracapsular Fractures of the Proximal Femur – is the Dynamic Hip Screw the Prevailing Gold Standard?

**DOI:** 10.2174/1874325001711011213

**Published:** 2017-10-31

**Authors:** Joshua Jacob, Ankit Desai, Alex Trompeter

**Affiliations:** 1 Orthopaedic Specialty Registrars, Ashford and St. Peter’s Hospital NHS Foundation Trust, Chertsey, UK; 2 Consultant Orthopaedic Trauma Surgeon, St. George’s Healthcare NHS Trust, London, UK

**Keywords:** Extracapsular, Fracture neck of femur, Management, Dynamic hip screw

## Abstract

Currently, approximately half of all hip fractures are extracapsular, with an incidence as high as 50 in 100,000 in some countries. The common classification systems fail to explain the logistics of fracture classification and whether they all behave in the same manner. The Muller AO classification system is a useful platform to delineate stable and unstable fractures. The Dynamic hip screw (DHS) however, has remained the ‘gold standard’ implant of choice for application in all extracapsular fractures. The DHS relies on the integrity and strength of the lateral femoral wall as well as the postero-medial fragment. An analysis of several studies indicates significant improvements in design and techniques to ensure a better outcome with intramedullary nails. This article reviews the historical trends that helped to evolve the DHS implant as well as discussing if the surgeon should remain content with this implant. We suggest that the gold standard surgical management of extracapsular fractures can, and should, evolve.

## 1 INTRODUCTION

A proximal femoral fracture refers to any fracture of the femur between the articular surface of the hip joint, and a point 5 cm distal to the distal part of the lesser trochanter. With the exception of fractures of the femoral head, these injuries are divided into two groups by their relationship to the capsular attachments of the hip joint. Intracapsular fractures occur proximal to the point at which the hip joint capsule attaches to the femur. Extracapsular fractures occur distal to the hip joint capsule. If the fracture line extends below the lesser trochanter the term “subtrochanteric” fracture is used.

The optimal implant for the surgical treatment of extracapsular hip fractures remains a contentious matter, with opinion divided over dynamic hip screw (DHS) devices and intramedullary nails (IMN). A 2005 Cochrane review compared a variety of implants and found no superiority in performance of intramedullary nails over the DHS and recommended the DHS on the basis of a lower incidence of complications by comparison [1]. Since then, however, the implant design of IMN has evolved substantially and a body of evidence supporting their use in certain situations is building.

The understanding of fracture stability is paramount in decision-making and the choice of implant. Not all fractures behave in the same manner and it is increasingly apparent that recognition of subtypes of extracapsular fractures is an important factor in implant selection. In this article, we address key operative decisions pertaining to these factors with evidence from current literature.

## 2 EPIDEMIOLOGY

Sir Astley Cooper’s 1824 treatise on dislocations and fractures noted that extracapsular fractures occurred infrequently in adults under 50 years of age by contrast with intracapsular ones [2]. Worldwide it is predicted that there will be over 3 million extracapsular hip fractures per annum by 2050 [3] and Hagino et al demonstrated that, at 50 years of age, their cohort had a 5% and 20% risk in men and women respectively of sustaining a hip fracture before they died [4].

As medical care advances and senescence increases, the clinical characteristics of patients change alongside and there are an increasing proportion of complex fracture patterns [5]. The management of complex injuries in less well patients carries an inevitable healthcare economic cost [6].

## 3 CLASSIFICATION – IS IT IMPORTANT?

While the distinction between intra- and extracapsular fractures is generally agreed, as are their different prognoses and management, such clarity does not exist for fractures of the proximal femur [7, 8]. For a classification system to be useful, it must be reliable, reproducible, relevant and informative and, while several systems have been described by which these fractures may be classified, none have fully satisfied all these criteria [9-12]. Broadly, as we have two types of implants available to us, any classification system should guide which of these implants is chosen and inform prognosis. The AO / OTA classification of long bone fractures has gained widespread acceptance and features prominiently in the literature [11]. Whilst its interobserver reliability is higher than other classifications in the area, it is still variable and, more crucially, it arguably does not provide adequate inforamtion on stability of the fracture [13].

Stability is a characteristic which can only be fully assessed in a reduced fracture. Stable fractures have cortical contact without a gap medially or posteriorly, preventing varus or procurvatum displacement. The AO/OTA 31A1 to 31A2.1 subtypes are typical of this. The 31A2.2 to 31A3.3 subtypes are progressively more unstable, with a loss of the medial buttress which, even if the head and shaft fragments are reduced relative to each other, will confer instability and the risk of varus collapse. While the 31A2.1 fracture also involves the medial buttress, the size of the fragment is sufficiently small that it is of little clinical consequence. The 31A3 subgroup exhibits loss of the lateral wall, including the reverse obliquity pattern, which render the fractures inherently unstable.

## 4 WHAT ARE WE HOPING TO ACHIEVE WITH THE IMPLANTS?

Regardless of the fracture pattern, the aims of surgery are the same – to restore the anatomy of the proximal femur [14], using a stable fixation device that would allow the patient to bear weight, with minimal soft tissue trauma and the least amount of physiological insult to the patient [14].

## 5 ARE INTRAMEDULLARY NAILS THE CORRECT IMPLANT FOR A2 AND A3 FRACTURES? 

Sahota et al’s 2007 systematic review found a higher incidence of complications with an intramedullary device than with the sliding hip screw [15]. They also noted insufficient evidence to detect a difference between individual types of intramedullary nails, and therefore advocated that newer designs should be evaluated against the current “gold standard” of a sliding hip screw. While these results are important, it should be noted that they included all extracapsular fracture patterns and hence do not address this specific clinical question adequately.

When considering 31A2 and 31A3 fractures, contemporaneous evidence is less equivocal and improved mobility and time to re-mobilization has been demonstrated, albeit not universally [16]. Lower rates of transfusion, shorter operative duration and reduced radiation exposure from image intensification have also been demonstrated.

Palm et al reported a 22% re-operation rate in patients undergoing DHS surgery in the context of a fractured lateral femoral cortex, by comparison with 3% in those with an intact wall. Of the lateral wall fractures, 74% were reported to be iatrogenic [17]. Gotfried et al’s series of 24 patients with post-operative collapse of fixed intertrochanteric hip fractures noted that this complication followed fracture of the lateral wall in every instance, underlining that not only should fractures involving the lateral wall be nailed, but also those which may involve it during the operation [18]. The risk of cut-out of the femoral neck screw may also be reduced by the use of an intramedullary device, due to the decreased moment arm and resistance to varus collapse [19]. This becomes increasingly important in 31A2.2 and 31A2.3 fractures, where loss of the posteromedial buttress results in a far greater cantilever load being applied to the implant.

When considering the choice of implant, one must also be mindful of any prevailing guidelines – in England, the National Institute for Health and Care Excellence’s guideline 124 advises a DHS for fractures as distal as and including the level of the lesser trochanter, in effect all 31A2 fractures [20].

## 6 SHORT OR LONG NAILS?

No evidence exists to demonstrate superiority of long intramedullary nails over their shorter counterparts [21]. They have similar complication profiles, including peri-prosthetic fracture, cut-out, avascular necrosis of the femoral head and symptomatic leg length discrepancy. Short nails, however, have shorter operative duration due to distal locking with the aid of an aiming jig [22]. The incidence of peri-prosthetic fracture seen with early-generation nails has now diminished as nail design has evolved, encompassing changes such as fluted tips, more proximal placement of the distal locking bolt and the use of different alloys.

Long nails are particularly useful in extracapsular fractures with sub-trochanteric extension and in pathological proximal femoral fractures. Trochanteric-entry nails are associated with an acceptable rate of peri-operative complications and favourable functional outcomes [23]. With long nails there exists a risk of perforation of the anterior cortex of the femur distally, at the tip of the nail, due to a mismatch of the radius of curvature. This is generally smaller in modern designs (114-120 cm) than in earlier generations of femoral nails (up to 300 cm) [24].

## 7 ARE ALL FEMORAL NAILS THE SAME?

A cephalomedullary femoral nail for extracapsular proximal femoral fractures differs from the reconstruction nail used for diaphyseal fractures. The proximal geometry and biomechanical principles differ, with a proximal femoral fracture nail filling the trochanteric region and acting to resist displacement forces, thereby preventing medial slide of the femoral shaft. It is thicker, necessitated by accommodating a large femoral neck compression screw or similar, and uses a trochanteric entry point. Since it is principally designed for proximal fractures, diaphyseal reaming is not required [25-27]. A reconstruction nail, by contrast, is typically smaller in diameter proximally, offering two 6.5mm or equivalent reconstruction screws into the femoral head. They are often slightly straighter, with less lateral bend in the trochanteric region, and they tend to be isthmic-bearing and thus require a reamed fit. They are not recommended for use in proximal femoral extracapsular fractures.

## 8 ARE ANY NEW TECHNIQUES AVAILABLE TO ME?

There have been several attempts to augment the biomechanical strength of both the DHS and intramedullary nails by using polymethacrylate (PMMA) preparations. In cadaveric studies, cement augmentation increased load to failure and minimized cut-out in both the DHS and intramedullary constructs [28]. This finding has been reproduced in patients with 31A2 fractures undergoing DHS surgery [29]. Recent studies using intramedullary nails have also shown that cement augmentation enhanced the implant anchorage within the head-neck fragment and conferred good functional results [30]. A robust, multi-centre, prospective trial concluded that augmentation with calcium phosphate cement in unstable trochanteric fractures provided a modest reduction in pain and a slight improvement in the quality of life when compared with the DHS alone [31].

Newer designs of plate, including pre-contoured locking plates designed for unstable A3 fractures, have been brought to market [32]. Recent innovations also include expanding femoral head screws, which are suggested to reduce cut-out, and biological membranes providing a scaffold for bone regeneration are emerging from translational work with veterinary fracture surgery.

## CONCLUSION

The ideal method of fixation is dependent on the stability of the fracture. “Simple” two part (A1 to A2.1) fractures can be adequately stabilised with the erstwhile “gold standard“ DHS implant, however, we have shown that this is not the ideal fixation for fractures classified A2.2 and above. The additional advantages of smaller incisions, shorter operating times, lower blood loss and transfusion requirements, and the avoidance of reaming, promote the current generation of intramedullary nails to be the gold standard in complex extracapsular hip fractures. The gold standard surgical management of extracapsular fractures can, and should, evolve.
